# A proteome view of structural, functional, and taxonomic characteristics of major protein domain clusters

**DOI:** 10.1038/s41598-017-13297-0

**Published:** 2017-10-27

**Authors:** Chia-Tsen Sun, Austin W. T. Chiang, Ming-Jing Hwang

**Affiliations:** 1 Institute of Biomedical Informatics, National Yang-Ming University, Taipei, 112 Taiwan; 2 Institute of Biomedical Sciences, Academia Sinica, Taipei, 115 Taiwan

## Abstract

Proteome-scale bioinformatics research is increasingly conducted as the number of completely sequenced genomes increases, but analysis of protein domains (PDs) usually relies on similarity in their amino acid sequences and/or three-dimensional structures. Here, we present results from a bi-clustering analysis on presence/absence data for 6,580 unique PDs in 2,134 species with a sequenced genome, thus covering a complete set of proteins, for the three superkingdoms of life, Bacteria, Archaea, and Eukarya. Our analysis revealed eight distinctive PD clusters, which, following an analysis of enrichment of Gene Ontology functions and CATH classification of protein structures, were shown to exhibit structural and functional properties that are taxa-characteristic. For examples, the largest cluster is ubiquitous in all three superkingdoms, constituting a set of 1,472 persistent domains created early in evolution and retained in living organisms and characterized by basic cellular functions and ancient structural architectures, while an Archaea and Eukarya bi-superkingdom cluster suggests its PDs may have existed in the ancestor of the two superkingdoms, and others are single superkingdom- or taxa (e.g. Fungi)-specific. These results contribute to increase our appreciation of PD diversity and our knowledge of how PDs are used in species, yielding implications on species evolution.

## Introduction

Proteins are formed by modules, commonly referred to as domains, linked together in a polypeptide chain. As a protein domain (PD) can be mutated, duplicated, deleted, or transferred from one species to another during evolution, they have been used as a unit to study not only protein structure and function, but also protein evolution. With the increased capability of technology to sequence genomes of various species, proteome-based bioinformatics studies have become commonplace^1,2^. For example, trees of life constructed using genome-wide PD content, i.e. properties of PDs such as their occurrence, abundance, and organization in the proteome of organisms, have been shown to be comparable with phylogenies derived by conventional methods, which usually rely on comparing sequences of a certain set of genes^3,4^. Furthermore, by analyzing the PD content of proteomes of various species, such studies can also reveal the origin and evolutionary history of PDs^5–10^ and identify those that seem to be used only by certain taxa, such as Bacteria^11–13^, that, for example, could then provide useful targets for the development of drugs against microbial pathogens^14,15^.

These post-genomic analyses are testaments to the wealth of knowledge that can be mined by interrogating the relationship between PDs and their species usage. Here, we present the results of a simple approach to dissecting this relationship, in which we carried out a bi-clustering analysis on a two-dimensional (2D) PD-species matrix in which one element was the presence (coded as unity) or absence (coded as zero) of a specific PD in a specific species. As its name indicates, in essence the bi-clustering analysis simultaneously considers the similarity of species occurrence between any two PDs (represented by two vertical vectors in the 2D matrix) and similarity of PD use between any two species (two horizontal vectors) based on the patterns of ones and zeros in these matrix vectors. For this analysis, we collected a total of 6,580 unique PDs from a total of 2,134 species (133 Archaea, 1,653 Bacteria, and 348 Eukarya) for which a fully sequenced genome (hence a complete set of PDs) is available. This represents one of the largest PD-species matrices analyzed to date^16,17^, especially when viruses are excluded from consideration.

Unlike the studies reported by Caetano-Anolles and colleagues^9^ in which the abundance of PDs in species was coded into 21 alphabets and a parsimonious path was used to define the evolutionary history of PDs, the aim of the present work was to identify PD clusters that were defined by similarity of species usage and characterize these in terms of species-related protein function and structure. Previously, PDs have been clustered according to similarity in their three-dimensional (3D) structure in several structure classification databases, such as SCOP^18^ and CATH^19^, which have proven to be very useful for structural bioinformatics research. In contrast, we used the 3D classifications of CATH and the functional annotations of Gene Ontology (GO)^20^ to characterize PD clusters that were defined by similarities of their species usage. Our results revealed several prominent PD clusters associated with specific classes of organisms, as well as characteristic 3D architectural design and molecular and cellular functions. Our results therefore provide new perspectives on the relationship between species usage and the structure and function of proteins.

## Methods

### Data

The taxonomical classifications of 2,134 species, each with a fully sequenced genome, were retrieved from UniProt^21^. Note that these species did not include viruses. For these species, 6,580 unique PDs and the three categories of Biological Process (BP), Cellular Component (CC), and Molecular Function (MF) of their functional annotation in Gene Ontolog (GO)^20^ and the four levels of Class (C), Architecture (A), Topology (T), and Homologous superfamily (H) of their 3D structure classification by CATH^19^ were downloaded from InterPro^22^. Note that InterPro – an expert curated PD database that provides comprehensive and integrative annotations for PDs – was used here to avoid potentially conflicting PD definitions. No other criteria were used to select these species (genomes) and all the protein domains classified as “Domain” in InterPro v42.0 (2014 version) were used, as we aimed to include as much data as possible in our analysis.

### Bi-clustering analysis

The information about the presence or absence of each of the 6,580 PDs in each of the 2,134 species yielded a 2,134 × 6,580 2D matrix. In this matrix, each of the 2,134 species rows is a horizontal vector of 6,580 elements, each of the 6,580 PD columns is a vertical vector of 2,134 elements, and every element in the matrix is either 1 or 0, representing, respectively, the presence or absence of a specific PD in a specific species.

Bi-clustering analysis (also known as co-clustering analysis) was then performed on the binary matrix using Generalized Association Plots (GAP), a tool for matrix visualization and clustering analysis^23^. The Jaccard similarity coefficient^24^ is used to compute the distance (i.e. similarity) between any two matrix rows (i.e. species) or between any two matrix columns (PDs), since this coefficient is especially suitable for measuring the similarity between sparse binary sets, which is the case in the PD-species matrix constructed here. During the clustering, rows of the matrix were moved up or down and columns moved left and right, such that similar rows (i.e. species with similar usage of PDs) and similar columns (PDs with similar patterns of species usage) would be clustered together as much as possible. This was achieved by transforming the PD-species matrix into a symmetric Robinson correlation matrix^25^ and optimizing it using an Elliptical seriation scheme^23^, which is very effective in identifying global and transitional clustering patterns^26^. Using a cutoff for the Jaccard similarity coefficient of 0.5, optimization resulted in eight PD clusters, which covered 4,019 PDs (61%), leaving 2,561 PDs (39%) scattered around the matrix that could not be assigned to any of the eight clusters without lowering the cutoff. The cutoff of 0.5 was chosen such that one of the 8 resultant PD clusters would cover as many as possible of the PDs encoded in a set of persistent genes reported by Acevedo-Rocha *et al*.^27^.

### GO and CATH analysis

An enrichment test based on the hyper-geometric distribution of GO terms^28^ was carried out for each of the eight PD clusters derived from the bi-clustering analysis. The enriched GO terms (p value < 0.05) in a studied PD cluster were then fed into the REViGO program^29^ to retrieve a set of representative GO terms that could be visualized using GO TermLogo (http://www.wordle.net/) as well as in a concentric GO pie showing their composition of the three GO categories (BP, CC, and MF). For the CATH structural analysis, we drew a CATH pie^19^ to display, within a given PD cluster, the percentage of PDs with a given structural category on each of the four levels of the CATH classification.

## Results

### Overview of the bi-clustering analysis results

**Figure 1:**
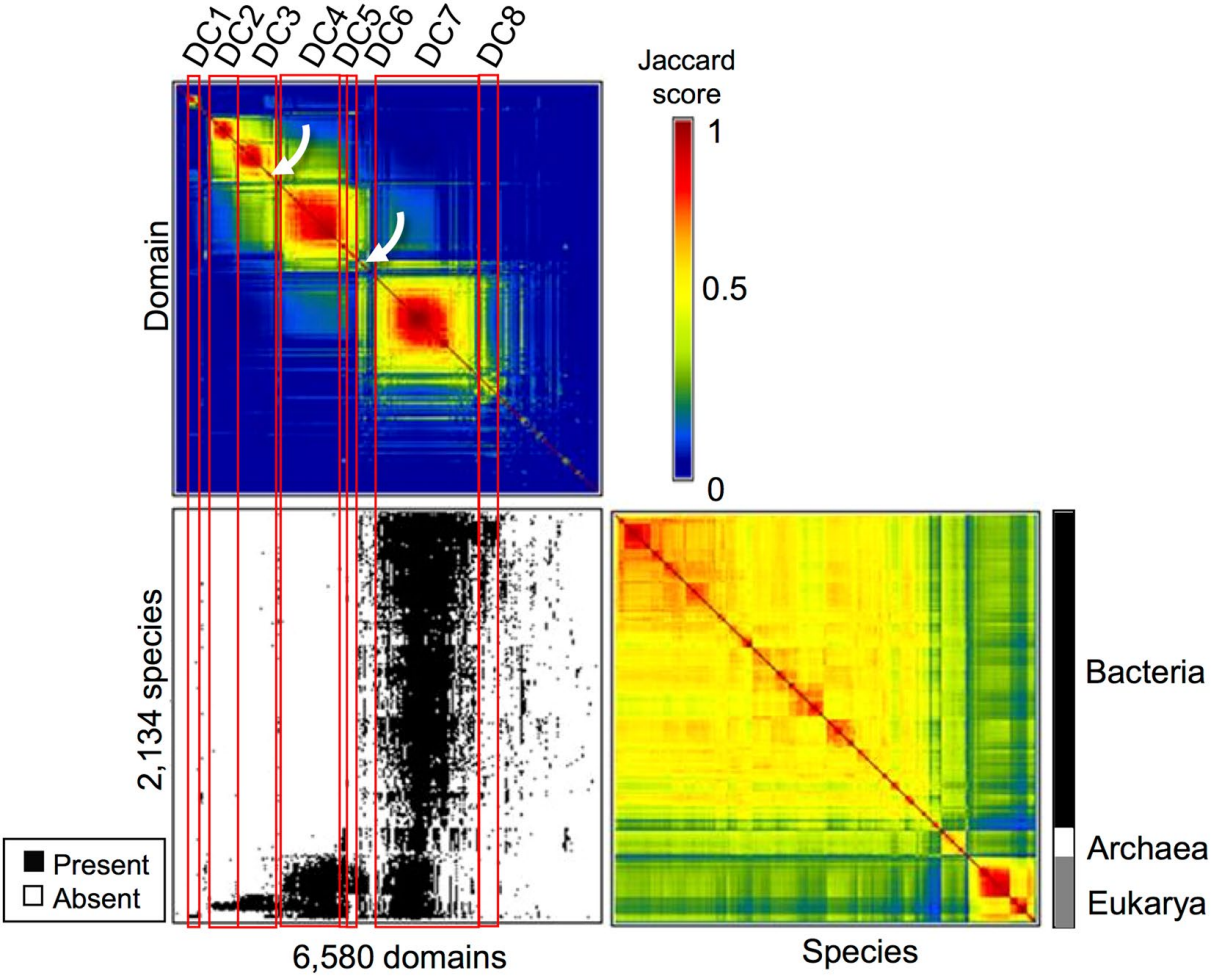
An overview of the GAP results. In this figure, there are three matrices: 1) The species-domain matrix (bottom left); a black dot indicates a domain is present in a species and a white dot that it is absent; 2) the domain-domain matrix (top left); when domains are present in certain species and absent in the rest, these domains will be clustered together, as indicated by warmer colors in the color scheme of the Jaccard similarity score. The 8 major domain clusters found using a Jaccard coefficient cutoff >0.5 (see Methods) are indicated. The arrows point to a small group of domains mentioned in the Discussion; 3) the species-species matrix (bottom right); when species use domains in a similar way, these species will be clustered together, as indicated by the warmer colors in the color scheme of the Jaccard similarity score (e.g. Eukarya species will be clustered together and not with Bacteria, as the domain present/absent patterns of Eukarya species are different from those of Bacteria).

Figure 1 is a standard GAP output of the bi-clustering analysis results. The bottom left panel shows the final PD-species presence/absence matrix after rearranging the rows and columns of the matrix to maximize the optimization score, the top panel is a heat map of the resulting PD-PD matrix showing the clustering of similar PDs (in warm colors), and the bottom right panel shows the species-species heat map generated by optimization. When the Jaccard coefficient was set at >0.5 (see Methods), eight PD clusters were seen, as indicated by the red vertical lines on the PD-PD map and the labels at the top (DC1-DC8). In addition, the species usage of these PD clusters can be seen in the PD-species matrix map, although not all dots are visible due to the low resolution of the map. Thus, for example, members of the largest PD cluster, DC7, are clearly used by species in all superkingdoms, while, in contrast, PDs in the first four clusters (DC1 to DC4) seem to be predominantly found in Eukarya species.

Note that species were cleanly separated into the superkingdoms of Archaea, Bacteria, and Eukarya (indicated by the vertical bar to the right of the species-species heat map). Furthermore, major taxonomic divisions within the same superkingdom, such as between Euryarchaeota and Crenarchaeota or between Gram-positive and Gram-negative Bacteria, could also be observed (see Supplementary Fig. S1), lending further support to previous findings that PD content in fully sequenced genomes is largely sufficient to classify species taxonomically^3,4^. Although more detailed analysis would probably reveal taxonomy-distinguishing PDs, we focused mainly on characterizing the eight PD clusters.

### The eight PD clusters

**Figure 2:**
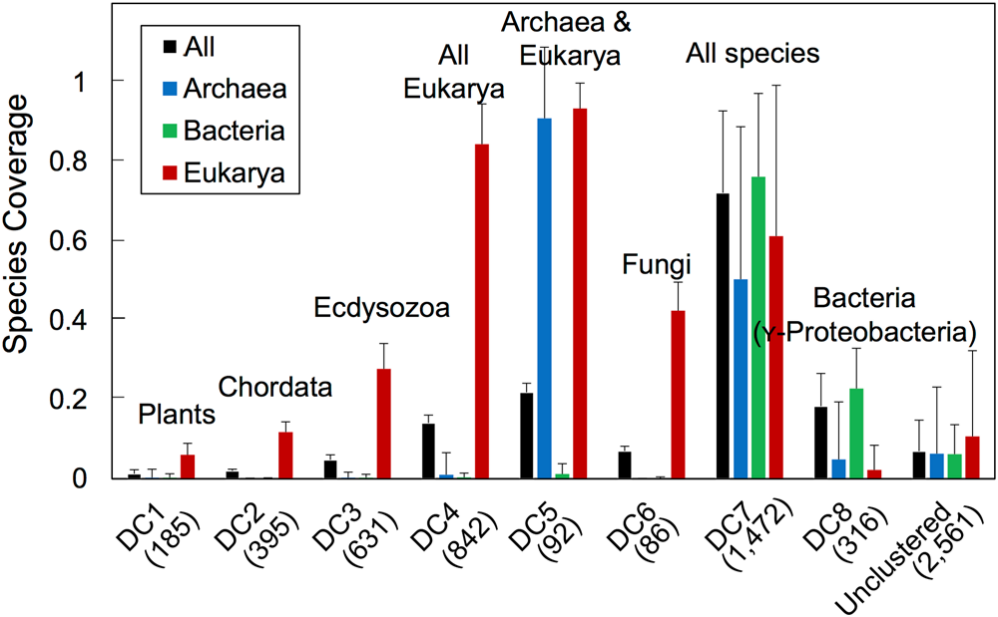
Species coverage of each of the eight PD clusters. A total of 133 Archaea species, 1,653 Bacteria species, and 348 Eukarya species were analyzed (see Methods). The Figure shows the averaged coverage of species in which a domain in a PD cluster (or the collection of unclustered domains) was found to be present; the bar indicates one standard deviation. Note that the number of completely sequenced plant genomes is smaller than that of other Eukarya taxa, resulting in the relatively small species coverage of DC1.

Table 1 shows the size (number of PDs) of each of the eight PD clusters and the taxa in which cluster members were mainly found. Figure 2 shows the species coverage (the fraction of the total number of species) in each of the three superkingdoms and in all superkingdoms combined in which members of a given PD cluster were found to be present, and confirms the observation from the low-resolution map in Fig. 1 that DC1-DC4 members were almost exclusively present in Eukarya species, while DC7 members were ubiquitous, although not present in every species, in all of the three superkingdoms of life. In addition, DC6, the smallest of the eight clusters, with only 86 PDs, was predominantly found in Eukarya species, while DC8, with 316 members, was predominantly found in Bacteria species, though with a low coverage of species. In contrast, DC5, another small cluster with 92 PDs, was found almost exclusively in two superkingdoms (Archaea and Eukarya), with almost no coverage of Bacteria species. The remaining 2,561 PDs (39%) did not share sufficient similarity with any of the above DCs or each other to be clustered (i.e. “unclustered”) and generally exhibited a low species coverage in any of the three superkingdoms.

**Table 1:** Size (number of domains) of the eight PD clusters and the taxa in which members were mainly found.

PD cluster	Number of PDs	Taxa
DC1	185	Viridiplantae
DC2	395	Chordata
DC3	631	Ecdysozoa
DC4	842	All Eukarya
DC5	92	Archaea & Eukarya
DC6	86	Fungi
DC7	1,472	Eukarya, Bacteria, and Archaea
DC8	316	γ-Proteobacteria

Further examination showed that, as indicated in Table 1, of the five Eukarya clusters DC1-4 and DC6, only DC4 had a wide coverage of all Eukarya species, as did the universal DC7 cluster and the Archaea-Eukarya bi-cluster DC5, while species coverage of DC1-3 and DC6 was highly restricted to specific taxa of the Eukarya superkingdom, namely, Viridiplantae (green plants) for DC1, Chordata (animals with a notochord) for DC2, Ecdysozoa (protostome animals) for DC3, and Fungi for DC6. The Bacteria cluster DC8 contained PDs that were found mainly in γ-Proteobacteria species, although with a marginal clustering score of similarity, as can be seen from the PD-PD heat map in Fig. 1; thus, despite the many clusters seen in the species-species heat map in Bacteria (Fig. 1), only one PD cluster, DC8, emerged from the bi-clustering analysis for this superkingdom.

### GO annotation of function

**Figure 3:**
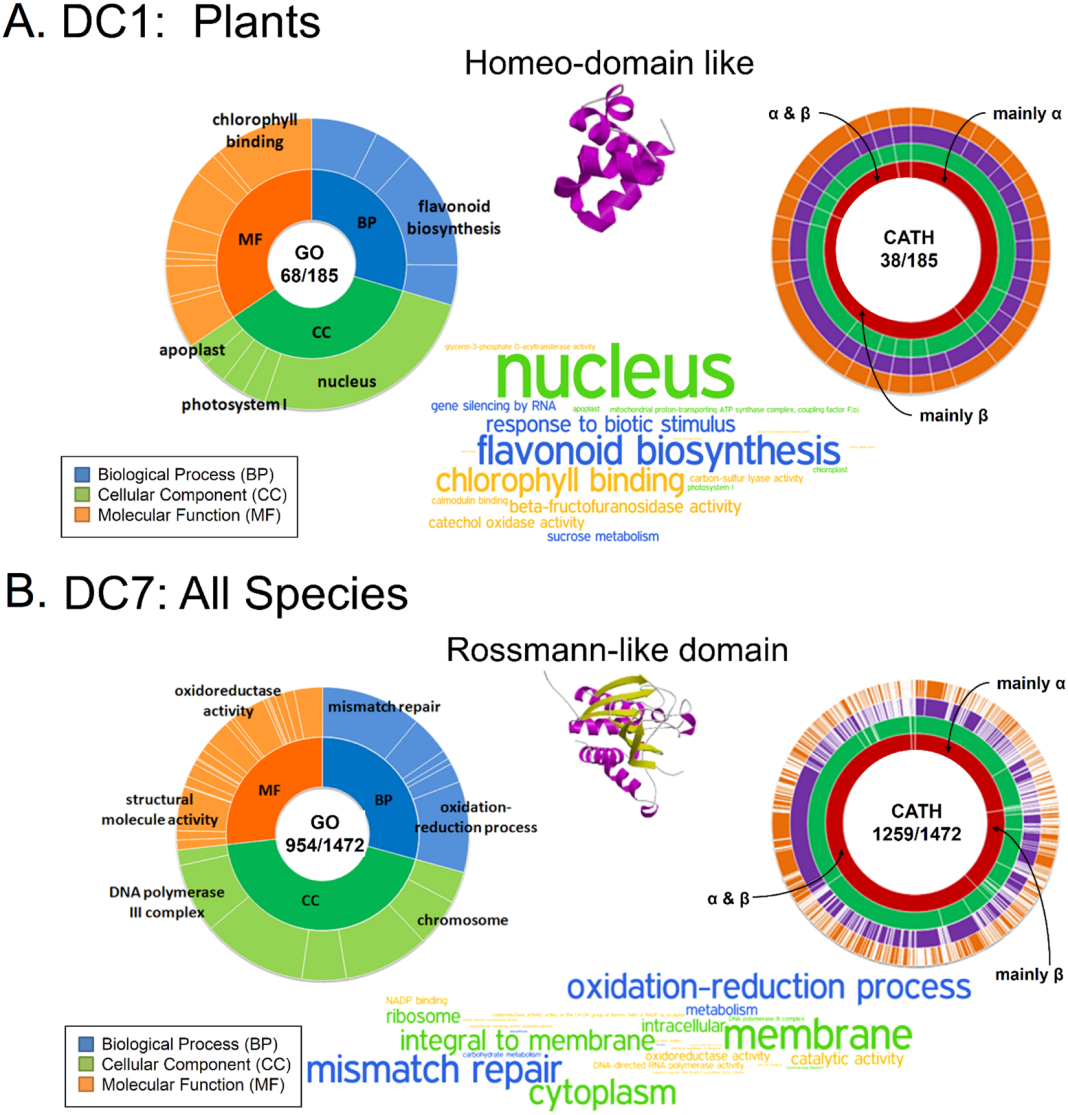
GO and CATH pies of the PD clusters DC1 and DC7. (**A**) DC1 (plants). (**B**) DC7 (all species). Left diagram: GO pie divided into 3 sections representing the 3 components of the GO annotation, biological process in blue, cellular component in green, and molecular function in orange. The outer circle displays the representative GO functions for the component, with white lines separating the different functions. Bottom center: the main representative GO functions are shown by GO TermLogo, with a bigger logo indicating a larger number of domains annotated with the logo function. Right diagram: CATH pie with four concentric circles, which represent, going from inside to outside, the 4 levels of the CATH classification, Class (red), Architecture (green), Topology (blue), and Homologous Superfamily (orange), with the categories (annotations) of each level separated by white lines. As in the GO pie, the number of PDs with an annotation was used to draw the divisions. The two numbers at the center of each GO and CATH pie are the number of PD with an annotation and the total number of PDs in the cluster. The 3D model shown between the two pies is an example of one of the PDs of the cluster.

Table 2 shows that between 25% and 65% of the PDs in a given cluster were annotated in GO and, of these, a high percentage, ranging from 67% to 86%, contained enriched GO terms. Examination of the enriched GO terms revealed some taxonomically-associated functions that seem to be cluster-specific. For example, as shown in the left panels of Fig. 3, PDs involved in photosynthesis were enriched in DC1, a green plant cluster, while PDs with basic cellular functions, such as DNA polymerase and oxidoreductase, were enriched in the universal cluster DC7. The “representative” GO terms generated by REViGO for the other six PD clusters, which are those labeled on the GO pie and shown as TermLogos in Supplementary Fig. S2, are also generally those functions used to characterize taxa; for example: 1) DC2 (Chordata) and DC3 (Ecdysozoa) contained many PDs with a function in the immune response, hormone activity, and growth factor activity, which are hallmarks of metazoan species^30^ and, consistent with DC2 and DC3 being separate clusters, Chordata (members of the Deuterostomia infrakingdom) and Ecdysozoa (members of the Protostomia infrakingdom) have evolved distinct proteins and mechanisms for these same functions^31–33^; 2) in DC4, the cluster almost exclusively found, and ubiquitous, in Eukarya species, one function that stood out was RNA polymerase II, a Eukarya-specific polymerase^34,35^; 3) the Archaea-Eukarya bi-cluster DC5 was dominated by the prefoldin complex, which is present in both Archaea and Eukarya though with a different oligomeric assembly between the two superkingdoms^36–38^; 4) DC6 was specific to fungi and its enriched functions included the synthesis of chitin, a main component of the unique fungal cell wall^39^; 5) DC8 was specific for γ-Proteobacteria, which are Gram-negative, and some of the enriched functions were associated with the type II secretion system, which is found in Gram-negative Bacteria and is involved in protein export^40^, and with glutathione biosynthesis, which is found primarily in Eukaryotes and Gram-negative Bacteria^41^.

**Table 2:** Statistics of annotated and enriched GO terms for the eight PD clusters.

PD cluster	PDs with a GO annotation	Annotated PDs with an enriched GO term
DC1 (Viridiplantae)	68/185 (37%)	49/68 (72%)
DC2 (Chordata)	160/395 (41%)	126/160 (79%)
DC3 (Ecdysozoa)	291/631 (46%)	194/291 (67%)
DC4 (All Eukarya)	387/842 (46%)	292/387 (75%)
DC5 (Archaea and Eukarya)	48/92 (52%)	41/48 (85%)
DC6 (Fungi)	22/86 (25%)	19/22 (86%)
DC7 (Archaea, Bacteria, and Eukarya)	954/1,472 (65%)	687/954 (72%)
DC8 (γ-Proteobacteria)	129/316 (41%)	98/129 (76%)

### CATH classification of structure

As shown in the right panels of Fig. 3 for DC1 and DC7 and of Supplementary Fig. S3 for the other six DCs, we used the number of PDs with a CATH annotation to draw a CATH pie consisting of 4 concentric circles representing the 4 levels of the CATH classification, which are, from the inside to outside, Class (red), Architecture (green), Topology (blue), and Homologous superfamily (orange), with white lines separating the different annotations. Thus, for example, the 38 DC1 PDs with a CATH annotation were divided into 15 (39%) in the mainly α class, 16 (42%) in the mainly β class, 6 (16%) in the α&β class, and 1 (3%) in the class with few secondary structures. Likewise, within the mainly α class, the PDs were divided into up to 5 Architectures (Orthogonal bundle, Up-down bundle, Alpha horseshoe, Alpha solenoid, and Alpha/Alpha barrel), each of which was divided into Topologies, which, in turn, were divided into Homologous Superfamilies. Note that, at any CATH level, some of the annotations may not be found in a given PD cluster, but, in general, a large cluster will need more white lines to separate its different annotations than a small cluster. For example, the universal cluster DC7 is significantly larger than DC1 (1472 PDs vs. 185 PDs; Table 1), and, consequently, has many more annotations and white separating lines.

Notwithstanding the bias in structure determination studies^42–44^, some interesting observations can be made from these CATH pie charts. For example, at the Class level in DC7, the highest proportion of PDs with a CATH annotation was seen in the α&β class (Fig. 3), suggesting that α&β is the most widely distributed structure class, since DC7 domains are ubiquitous in all three superkingdoms. This is consistent with studies suggesting that α&β structures are the most ancient of protein structures^9,45^. Similarly, though to a lesser extent, many of the PDs of DC5, the cluster for Archaea and Eukarya, were α&β structures (Supplementary Fig. S3). In comparison, only 9 (of 86) domains in DC6 (the fungi cluster) were annotated in CATH, and none was a β structure (Supplementary Fig. S3), possibly indicating a skewed representation in the database of 3D protein structures for fungi (discussed later).

Next, we examined the CATH annotations at the Architecture level for the three main Classes (mainly α, mainly β, and α&β). The architecture present at the highest frequency in the mainly α class was Orthogonal bundle (Supplementary Fig. S4), which is thought to be associated with protein stability^46^ and considered to be an ancient architecture of protein structures^9^. In the mainly β class, one notable architecture present at high frequency was the β-sandwich, as in the PKC-C2 domain, which is involved in targeting proteins to cell membranes and is known to be present in Eukaryotes^47^, in agreement with the architecture’s abundance in DC1-DC4 (Supplementary Fig. S5), which are Eukarya clusters (Fig. 2), though, interestingly, this architecture was absent in DC5, which has a high coverage of both Eukarya and Archaea species. In addition, β sheets are widely used in thermophiles^48^, and this is consistent with the relatively high proportion of single sheet architecture in DC5 (Supplementary Fig. S5). The β-barrel architecture occurred at a high frequency in DC7 and DC8 (Supplementary Fig. S5), in agreement with its being the most ancient architecture of barrels^9^ and with the observation that approximately 2–3% of the genes in Gram-negative bacterial genomes, the main species of DC8, encode β-barrel proteins^49^. In the α&β class, the 3-Layer (αβα) and 2-Layer architectures were most abundant (Supplementary Fig. S6). Many enzymes and nucleotide-binding proteins adopt the 3-Layer sandwich architecture, the oldest architecture of all proteins^9,45,50^, and more than 30 ribosomal proteins belonging to the 2-Layer sandwich architecture are found in many Archaea and Eukarya species, but not in Bacteria^51^, although other PDs with the 2-Layer architecture are present in Bacteria species (Supplementary Fig. S6).

Finally, Table 3 shows that, although there were different numbers of domains with a CATH annotation in the eight PD clusters, the majority of those annotated belonged to homologous superfamilies (H) containing PDs found in only one PD cluster, which echoes the observation of taxonomically-associated GO functions.

**Table 3:** Cluster-specific homologous superfamilies (Hs) in the eight PD clusters.

PD cluster	PDs with a CATH annotation	Unique Hs that are cluster-specific^a^
DC1 (Viridiplantae)	38/185 (21%)	22/32 (69%)
DC2 (Chordata)	142/395 (36%)	47/95 (49%)
DC3 (Ecdysozoa)	265/631 (42%)	85/177 (48%)
DC4 (All Eukarya)	356/842 (43%)	137/221 (62%)
DC5 (Archaea and Eukarya)	71/92 (77%)	27/57 (47%)
DC6 (Fungi)	9/86 (10%)	6/8 (75%)
DC7 (Archaea, Bacteria, and Eukarya)	1,259/1,472 (86%)	548/665 (82%)
DC8 (γ-Proteobacteria)	157/316 (50%)	74/130 (57%)

^a^Ratio (%) of cluster-specific homologous superfamilies to the total number of unique homologous superfamilies for PDs with a CATH annotation.

## Discussion

In this study, we showed that a simple bi-clustering analysis of species usage of PDs was able to reveal clusters of PDs with taxa-associated protein 3D structures and functions. Bi-clustering analysis of binary-transformed biological data has been shown to be fruitful (see, e.g., Ding *et al*.^52^). The bi-clustering method GAP was chosen here because, as noted in Methods, of its ability to identify both global and transitional clustering patterns^26^. Besides GAP, three other bi-clustering algorithms accessible from an R toolbox^53^ were also tried. However, these three methods were designed to find sub-matrices in gene expression data, and they would identify different and special patterns of the species-PD binary data (see Supplemental Figs S7 and S8) depending on the objective of their original design. Specifically, Bimax^54^ tends to find sub-matrices having all ones (“1”), resulting in 10 clusters each with few PDs that are present in all species of the cluster (size too small to be visible on the plot of Fig. S7), while Xmotifs^55^ tends to identify sub-matrices whose rows have the same state (i.e. either “1” or “0”) over a set of columns, yielding five groups in which protein domains are “absent” in the group’s species. While Plaid^56^ has been claimed to be one of the most flexible bi-clustering methods, a recent study showed that it is sensitive to noise^57^; in our case, it identified only three clusters that are dissimilar to the GAP-derived clusters. Although some of the special sub-matrices (i.e. PD clusters) identified by these other methods may turn out to bear biological significance, they require further analysis in future studies.

More than half of the PDs analyzed had no GO or CATH annotation (the second column of Tables 2 and 3), underscoring the incompleteness of GO and CATH annotations for sequence-derived PDs. However, despite the low coverage, our results showed a high percentage of enriched GO/CATH annotations (the third column of Tables 2 and 3), which means that these PDs are strongly associated with the annotated biological functions and 3D structures. Furthermore, a ~60% coverage was achieved by combining the two (Supplementary Table S1), suggesting that considering both structure and function together allows more PDs to be studied from a broader perspective by computational analysis such as the bi-clustering analysis used here. Although much has been revealed by studies that solely rely on protein sequence^3–9^ or structural information^10,46–51^, many questions about the complicated relationships between protein function, structure and species evolution remain unexplored. The dataset (Supplementary Table S2) of integrating PDs, functional and structural annotations, and species therefore provides valuable resources for future proteome research.

Our analysis identified eight taxa-associated PD clusters. Of these, only DC8 (specific to γ-Proteobacteria) did not cover a significant number of Eukarya species or a specific Eukarya taxa (Fig. 2). However, this is not surprising, since it is well-recognized that Eukarya species are more diverse than species of the other superkingdoms, as a rapid expansion in proteins occurred during the evolution of Eukarya species to allow them to adapt to the changing environments and lifestyles of the Proterozoic period^58^. Interestingly, in contrast to DC1–4 and 6 (Eukarya-specific) and DC8 (Bacteria-specific), none of the PD clusters was Archaea-specific. Further analysis showed that, of the 6,580 PDs analyzed, 48 were only found in various Archaea species, but these 48 had a low species coverage, and their species usages were not similar enough to form a cluster to represent Archaea. Furthermore, recent studies showed that multicellular metazoans’ evolution could be accounted for by different domain usage. For example, the rapid increase of diverse domains for cell-to-cell communications contribute to the development of higher life form of multicellular eukaryotes^59^. Interestingly, our further analysis (Supplementary Fig. S9) showed different distributions of multicellular species coverage for the eight DCs. Indeed, DC1-4&6 (Eukarya-dominated PD clusters) have high multicellular species coverage (>60%), but DC7-8 (the ancestral and Bacteria PD clusters) and unclustered PDs have low multicellular species coverage (<20%). All these results suggest that the different domain usage by species is closely linked to evolution mechanisms and contributes to the development of different life forms.

The only bi-superkingdom cluster was DC5, with a high species coverage of Archaea and Eukarya, but almost no coverage of Bacteria (Fig. 2). This suggests a close relationship between the two superkingdoms, evolution of which is usually interpreted by either of two scenarios: 1) Eukarya and Archaea are two distinct sister linages sharing a common ancestor, or 2) Eukarya have evolved from Archaea^60–62^. However, we note that, although the species tree from the bi-clustering analysis agreed well with the taxonomy separation of the three superkingdoms (Supplementary Figs S1 and S10), we made no attempt to include additional data necessary for reconstructing and optimizing a phylogenomic tree to support or dispute these evolutionary scenarios. Nevertheless, our analysis does indicate that DC5 comprises ancient PDs, such as prefoldin^36–38^ and proliferating cell nuclear antigen^63–65^, which have not been shown to be present in Bacteria. About 10% of DC6 (fungi) domains were annotated in CATH (Table 3), and most of these came from yeast (*Saccharomyces cerevisiae*), one of the most highly studied model organisms. However, more than 60% of PDs found in fungal species were, in fact, annotated in CATH (data not shown), suggesting that few fungal PDs are specific to fungal species. One example of the fungi-associated PDs in DC6 is the redox domain of the transcription factor YAP1, which is a central regulator that responds to oxidative stress in fungi^66^.

In the effort to find a ‘minimal genome’ required for cell survival that has potential applications in synthetic biology, the concept of ‘persistent genes’ has been proposed^67^. Previously, ‘persistent genes’, which are thought to encode functions essential for either continual production of progeny of a cell or cellular maintenance, stress responses, and repair, were defined as genes conserved in a majority of Bacteria genomes, as, for example, in the work of Acevedo-Rocha *et al*.^27^ who combined *in vitro* experiments and in silico analysis to identify 610 persistent genes in ~1000 Bacteria species. In the present study, the 1,472 members of the universal cluster DC7 can be considered as ‘persistent PDs’, because they were found in most living species (Fig. 2) while, as indicated by the analysis of the GO and CATH annotations, exhibiting activities of essential cellular functions and ancient structural architectures. An analysis of the 610 persistent Bacteria genes reported by Acevedo-Rocha *et al*. showed that they encode 776 unique PDs, of which only 38 (<5%) were not included in DC7 (data not shown), 35 of which were present in DC8, the γ-Proteobacteria cluster, and the remaining 3 could not be assigned to any of the eight PD clusters. These suggest that we have identified a set of persistent PDs in DC7, which roughly doubles the number of ‘persistent genes’ identified in Bacteria, that constitutes a core set of PDs representing essential functional and structural units that originated early in evolution and must be maintained in living organisms.

A closer look at the PD-PD heat map in Fig. 1 offers other interesting observations besides the identification of the eight main clusters described above: 1) a small group of PDs in the bottom-right corner of the DC3 (Ecdysozoa) cluster (indicated by an arrow on the map) appear to be somewhat distinct from the rest of the cluster and, interestingly, show a faint similarity (colored light blue) with DC1, the green plant cluster; 2) the Fungi cluster (DC6) appears to be more similar to DC4, the ubiquitous Eukarya cluster, than to DC5, the Archaea-Eukarya bi-cluster (yellowish vs. greenish colors); 3) a small group of PDs bridging DC4-6 to the universal cluster DC7 can be seen, which share some similarities (greenish colors) with DC4, 5, and 7, but much less so with DC6, the Fungi cluster. The significance of these additional observations awaits further evaluation, which may lead to the discovery of signature PDs with implications in the evolution of major lineages in the tree of life. Furthermore, some of the single taxa (e.g. Fungi or Bacteria)-specific PDs may prove to be useful targets for drug development against pathogens.

## Electronic supplementary material

Supplementary Information

Supplementary Dataset
