# Supplementary material for: A proteome view of structural, functional, and taxonomic characteristics of major protein domain clusters
Source: Sci Rep. 2017 Oct 27;7:14210. doi: 10.1038/s41598-017-13297-0 (PMC5660162; doi:10.1038/s41598-017-13297-0)
Supplement: Supplementary file 1 — Supplementary Information [file 41598_2017_13297_MOESM1_ESM.pdf]

A proteome view of structural, functional, and taxonomic characteristics of major protein domain clusters

Chia-Tsen Sun[1,2+], Austin W.T. Chiang[2+], and Ming-Jing Hwang[1,2*]

[1] Institute of Biomedical Informatics, National Yang-Ming University, Taipei 112, Taiwan
[2] Institute of Biomedical Sciences, Academia Sinica, Taipei 115, Taiwan.

[+] These authors contributed equally to this work
[*] Corresponding author

Email addresses:

CTS: cherry79722@gmail.com

AWTC: austin.chiang@gmail.com

MJH: mjhwang@ibms.sinica.edu.tw

Supplementary Information

Table S1. Coverage statistics of the eight PD clusters on their GO and CATH annotations.

Table S2. A full list of the 6,580 protein domains analyzed, with their PD cluster, species, and CATH and GO annotations.

Figure S1. Clusters of Archaea, Bacteria, and Eukarya species.

Figure S2. GO pies for the eight PD clusters.

Figure S3. CATH pie charts for the eight PD clusters – Class level.

Figure S4. CATH pie charts for the eight PD clusters – Architectures of the mainly α class.

Figure S5. CATH pie charts for the eight PD clusters – Architectures of the mainly β class.

Figure S6. CATH pie charts for the eight PD clusters – Architectures of the α&β class.

Figure S7. Visualization of bi-clustering results from different methods.

Figure S8. Coverage of species and domains from different bi-clustering methods.

Figure S9. Multicellular species coverage of each of the eight PD clusters.

Figure S10. Comparison of a conventional phylogenetic tree with GAP clustering tree.

Table S1. Coverage statistics of the eight PD clusters on their GO and CATH annotations

	**DC1**	**DC2**	**DC3**	**DC4**	**DC5**	**DC6**	**DC7**	**DC8**	**Mean**
**Number of PDs**	**185**	**395**	**631**	**842**	**92**	**86**	**1,472**	**316**	
% of PDs with only a GO annotation	24.3	23.5	19.0	19.4	9.8	19.8	7.9	16.5	**17.5**
% of PDs with only a CATH annotation	8.1	19.0	14.9	15.7	34.8	4.7	28.6	25.3	**18.9**
% of PDs with both GO and CATH annotations	12.4	17.0	27.1	26.6	42.4	5.8	56.9	24.4	**26.6**
GO + CATH (%)	44.8	59.5	61.0	61.7	87.0	30.3	93.4	66.2	**63.0**

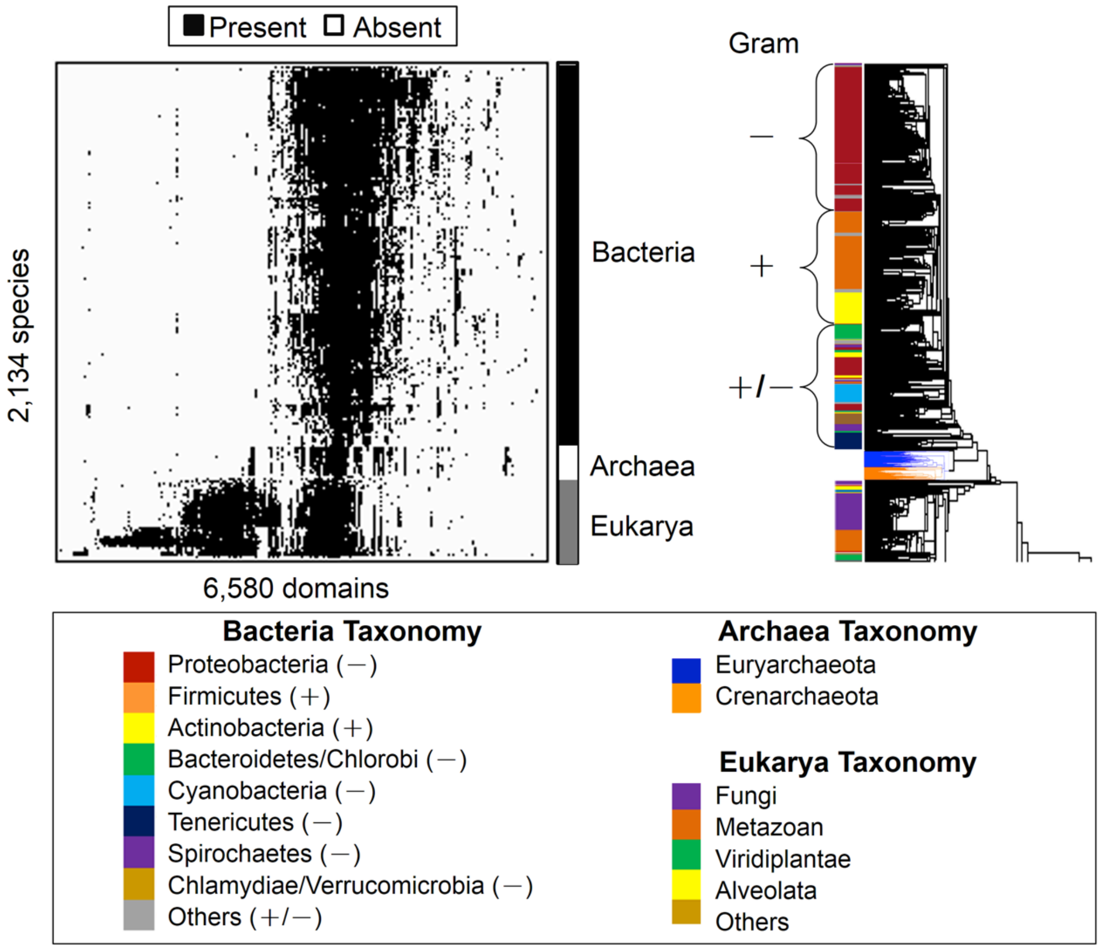

Figure S1. Clusters of Archaea, Bacteria, and Eukarya species. The GAP bi-clustering result shows that species from different superkingdoms (Archaea, Bacteria, Eukarya) do not mix and can be grouped separately into 3 clusters. Furthermore, within the Archaea species cluster, Euryarchaeota phylum species are separated from those of the Crenarchaeota phylum. The Bacteria cluster, with considerably more sequenced genomes, is the largest of the three species clusters. The clustering result shows that most of the Bacteria species in the same phylum cluster together, e.g. Gram-negative Bacteria (Proteobacteria, Bacteroidetes/Chlorobi, Cyanobacteria, Tenericutes, Spirochaetes, and Chlamydiae/Verrucomicrobia) are generally separated from Gram-positive Bacteria, such as Firmicutes and Actinobacteria. Furthermore, although some Bacteria, such as Proteobacteria and Firmicutes, were not clustered at the phylum level, they were clustered at the class level (data not shown).

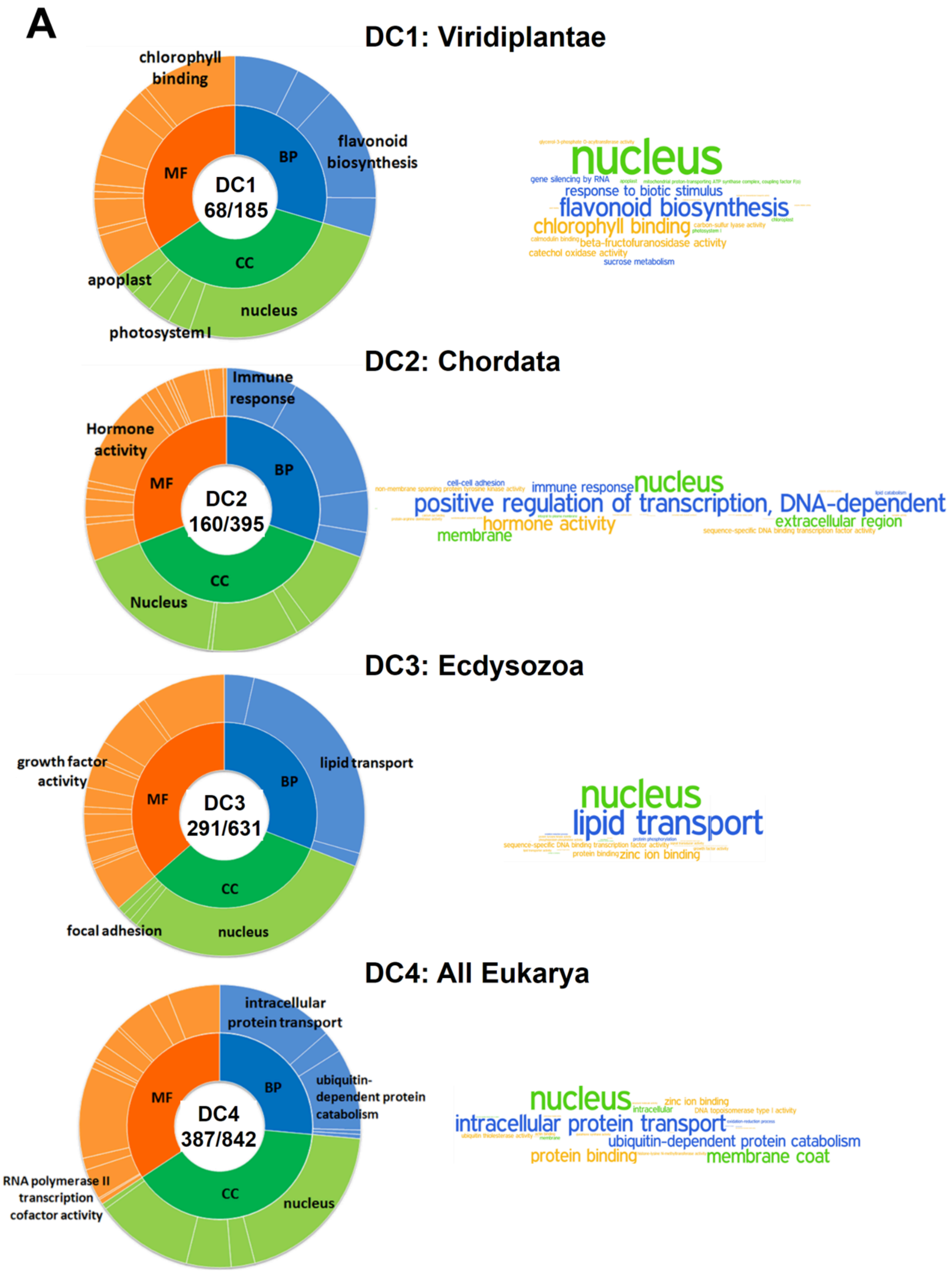
A
DC1: Viridiplantae
chlorophyll binding
flavonoid biosynthesis
MF
BP
CC
DC1 68/185
apoplast
photosystem I
nucleus
nucleus
response to biotic stimulus
flavonoid biosynthesis
chlorophyll binding
beta-fructofuranosidase activity
catechol oxidase activity
sucrose metabolism
gene silencing by RNA
DC2: Chordata
Immune response
Hormone activity
MF
BP
CC
DC2 160/395
Nucleus
cell-cell adhesion
immune response
nucleus
positive regulation of transcription, DNA-dependent
hormone activity
extracellular region
membrane
DC3: Ecdysozoa
growth factor activity
lipid transport
MF
BP
CC
DC3 291/631
focal adhesion
nucleus
nucleus
lipid transport
zinc ion binding
DC4: All Eukarya
intracellular protein transport
ubiquitin-dependent protein catabolism
MF
BP
CC
DC4 387/842
RNA polymerase II transcription cofactor activity
nucleus
nucleus
zinc ion binding
intracellular protein transport
ubiquitin-dependent protein catabolism
protein binding
membrane coat

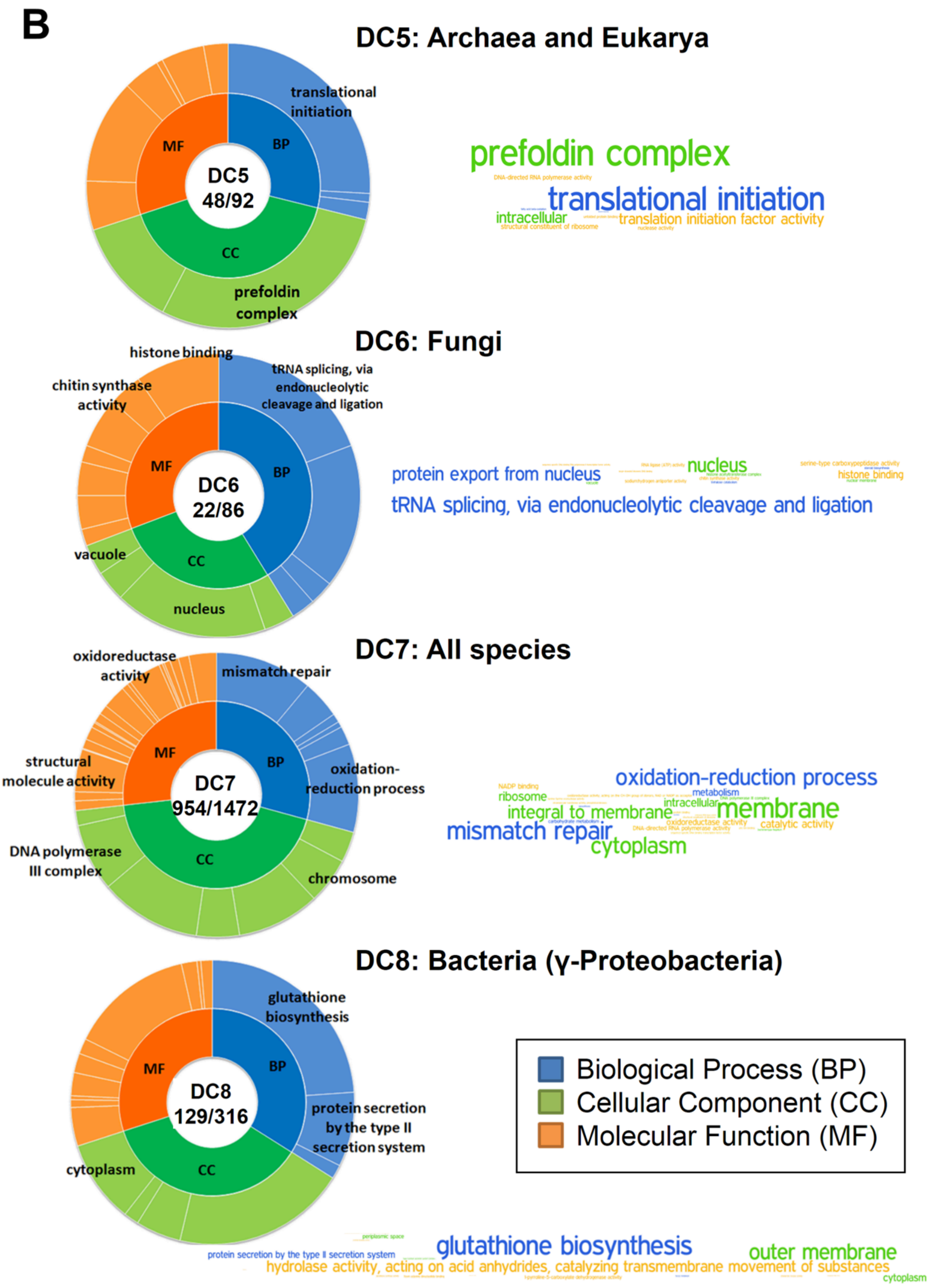

Figure S2. GO pies for the eight PD clusters. (A) DC1 to DC4; (B) DC5 to DC8. All notations and symbols are the same as those in Figure 3 in the main text. DC1 and DC7, already presented in Figure 3, are included here for completeness.

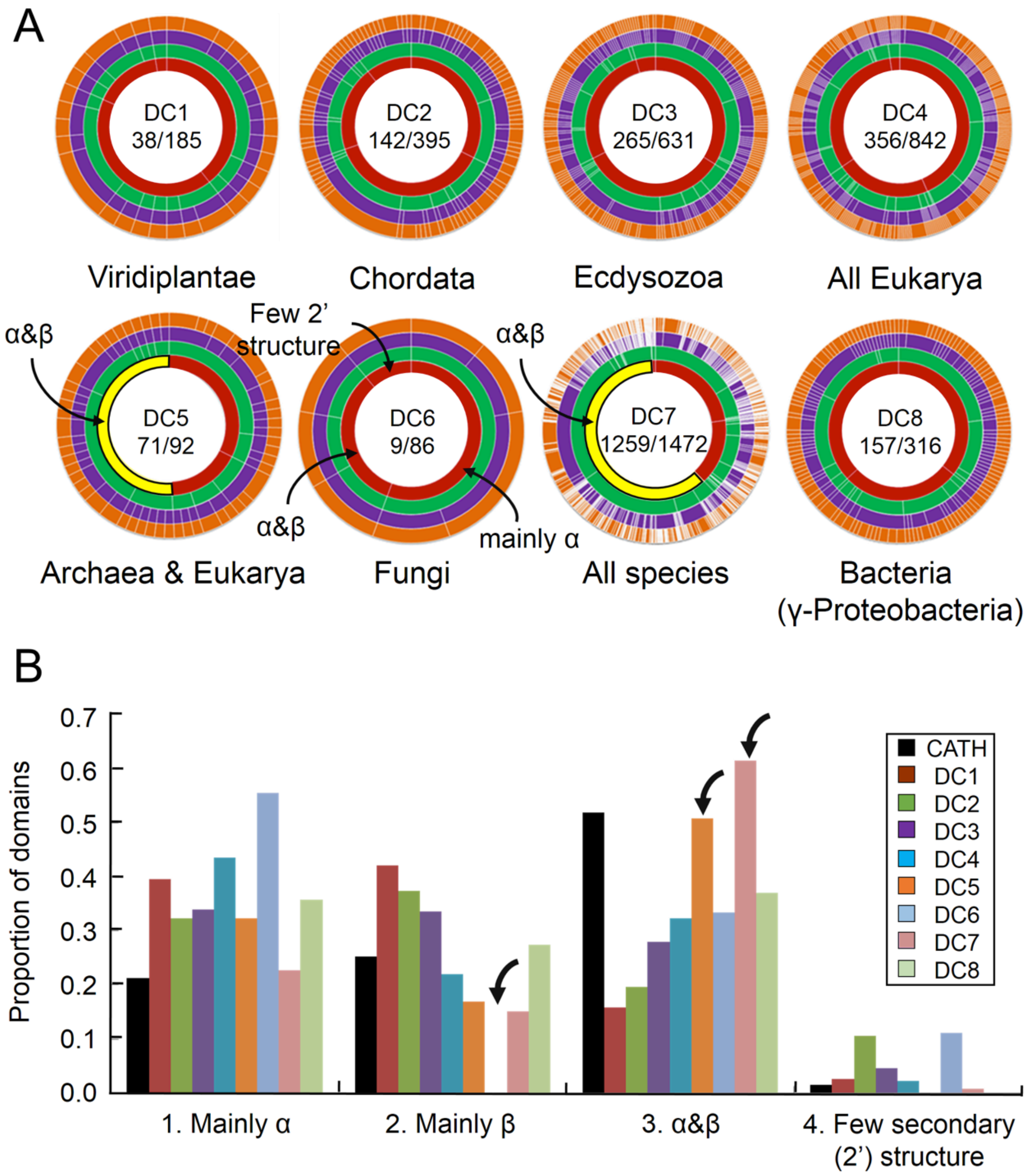

Figure S3. CATH pie charts for the eight PD clusters – Class level. (A) The pie charts represent the 4 levels of the CATH annotations (4 concentric circles) for each of the 8 protein domain clusters. The largest Class is highlighted in yellow for two examples, DC5 and DC7. (B) The bar charts show the proportion of the total PDs with an annotation found in the indicated DC cluster in the indicated Class. The black bars are the statistics for the entire CATH database. The bar height corresponds to the proportion of the divisions (separated by white lines) used in the pie. The arrows indicate data discussed in the main text.

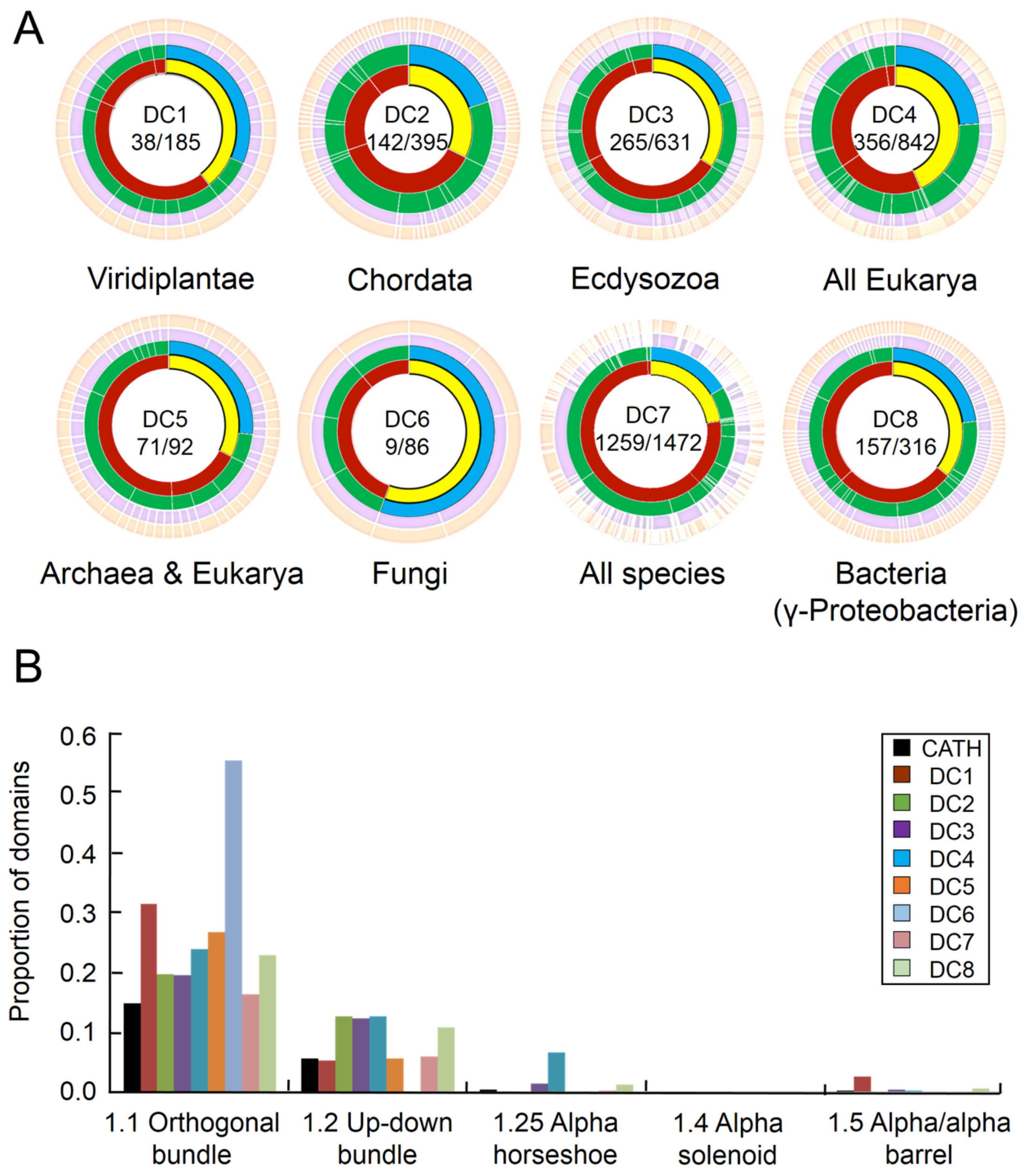

Figure S4. CATH pie charts for the eight PD clusters – Architectures of the mainly α class. The notations are the same as those in Figure S3. The largest Architecture of the mainly α class (yellow), Orthogonal bundle, is highlighted in blue. The bar heights in the bar chart correspond to the proportion of the pie chart divisions.

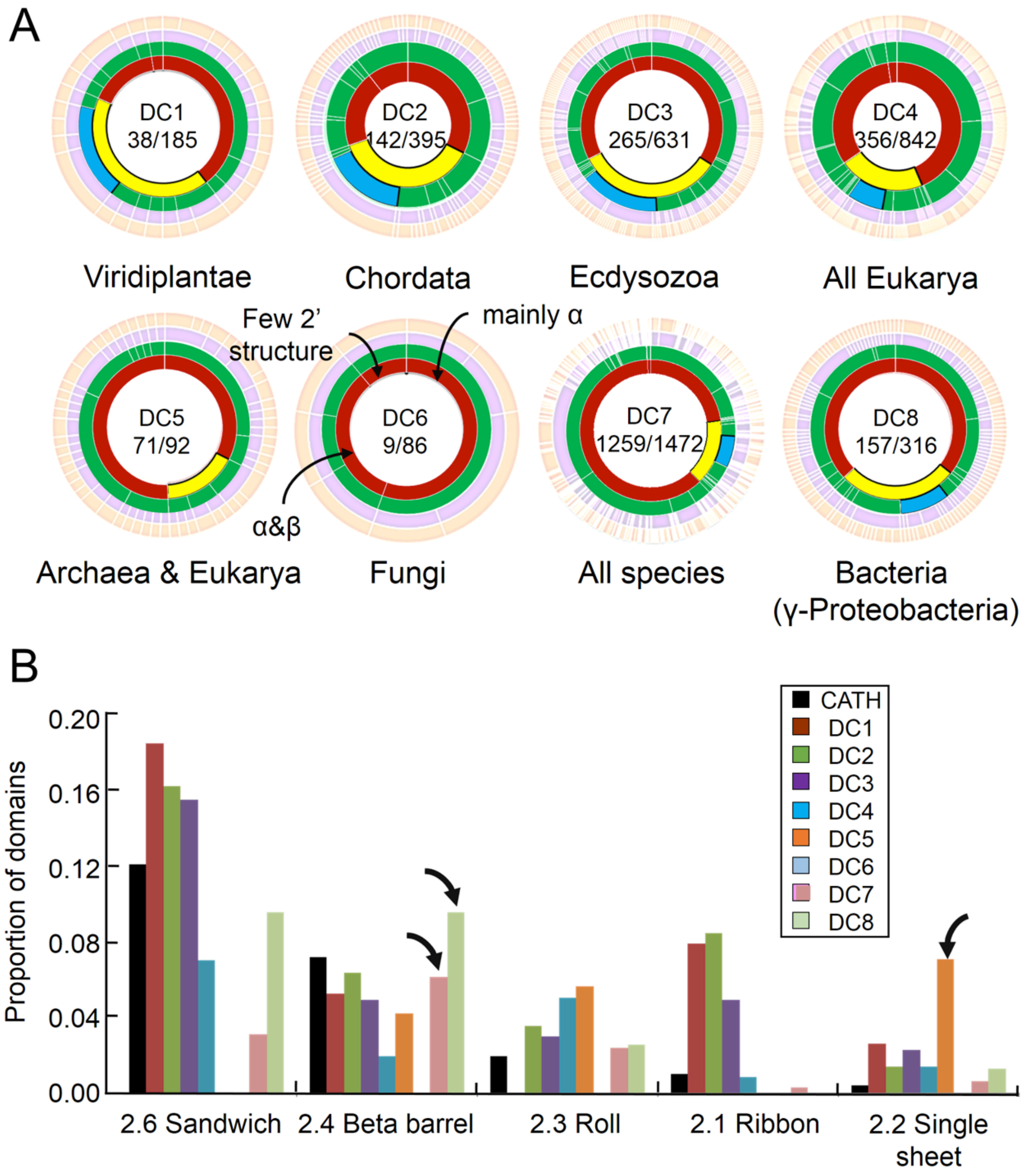

Figure S5. CATH pie charts for the eight PD clusters – Architectures of the mainly β class. The notations in the pie chart and bar chart are the same as those in Figure S3. The most abundant Architecture (β-sandwich) of the mainly β class (yellow) is highlighted in blue. Note that the Fungi cluster (DC6) does not have a mainly β-class structure annotated in CATH. The bar heights correspond to the proportion of the pie chart divisions. The arrows indicate data discussed in the main text.

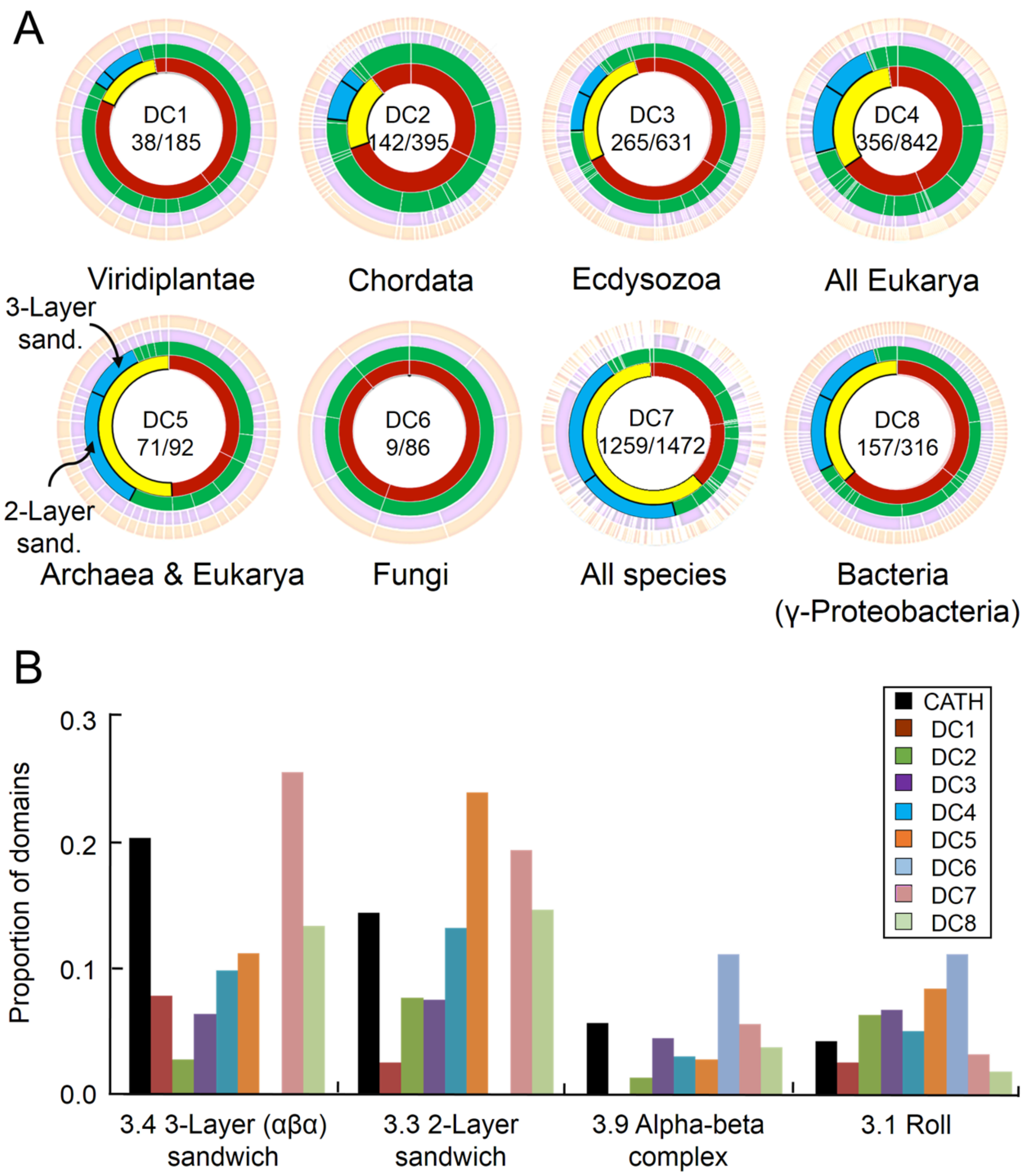

Figure S6. CATH pie charts for the eight PD clusters – Architectures of the α&β class. The notations in the pie chart and bar chart are the same as those in Figure S3. The two most abundant architectures (3-Layer and 2-Layer sandwich) of the α&β class (yellow) are highlighted in blue. The bar heights in the bar chart correspond to the proportion of the pie chart divisions.

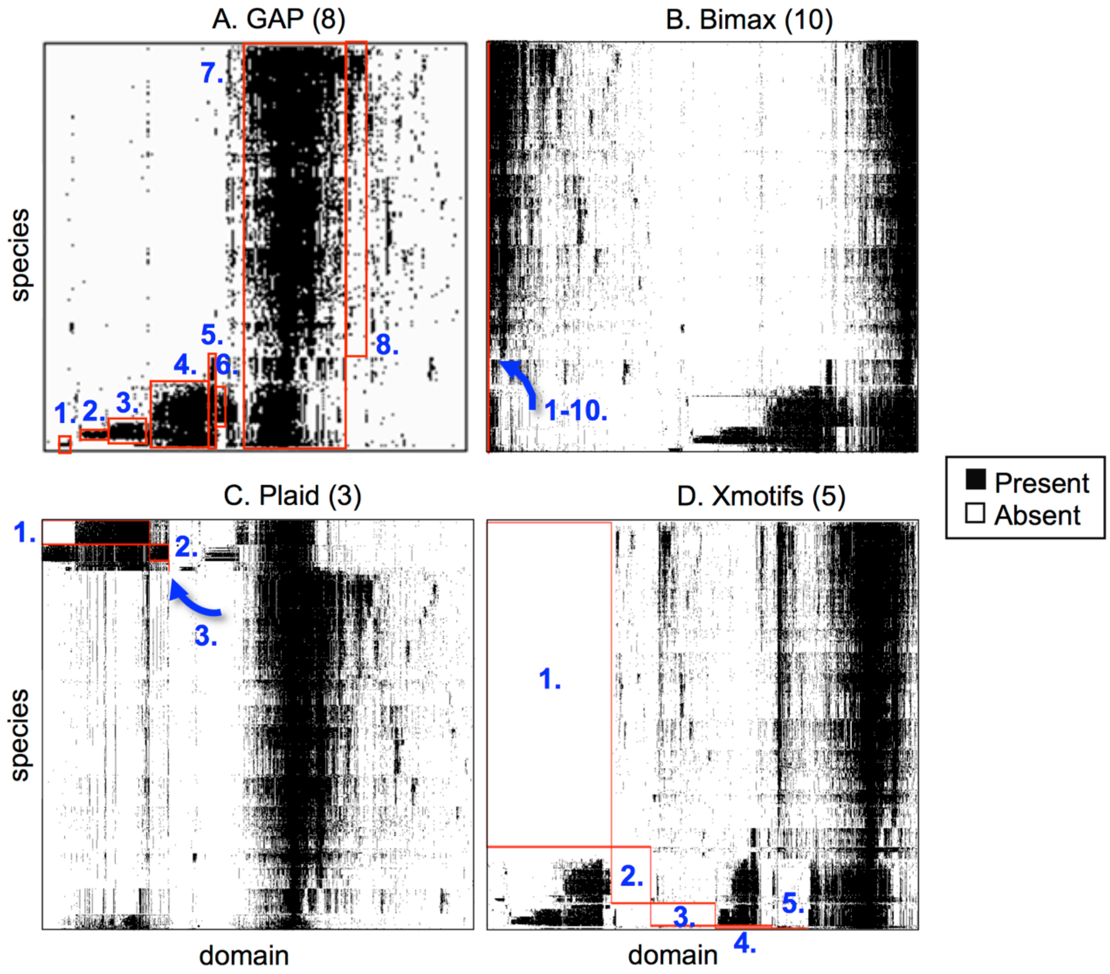

Figure S7. Visualization of bi-clustering results from different methods. A) GAP [1]; B) Bimax [2]; C) Plaid [3]; and, D) Xmotifs [4]. In parenthesis are the number of clusters identified by each method. The clusters (red rectangles) identified by each method were also labeled by blue numbers. The GAP plot was created by the GAP tool, and the others were created by the R package "biclust" (Kaiser and Leisch [5]). Note that these other methods were originally designed to find sub-matrices in gene expression data and different methods may identify different patterns in gene expression profiles. For example, Bimax [2] tends to find sub-matrices having all ones ("1"), resulting in 10 clusters each with few PDs that were present in all species of the cluster (size too small to be visible on the plot), while Xmotifs [4] tends to identify sub-matrices whose rows have the same state (i.e. either "1" or "0") over a set of columns, yielding, in our case, five groups in which protein domains were "absent" in the group's species.

A. Number of clusters resulted and their total coverage of protein domains and species

	GAP	Bimax	Plaid	Xmotifs
Cluster	8	10	3	5
Domain	4019	22	1937	4899
Species	2134	2134	264	2134

B. Venn diagram of species

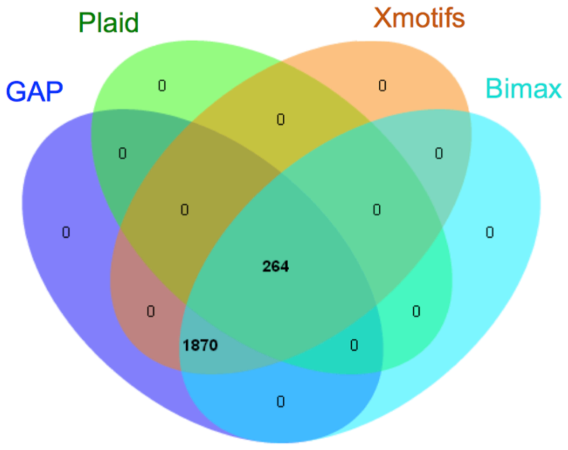

C. Venn diagram of domains

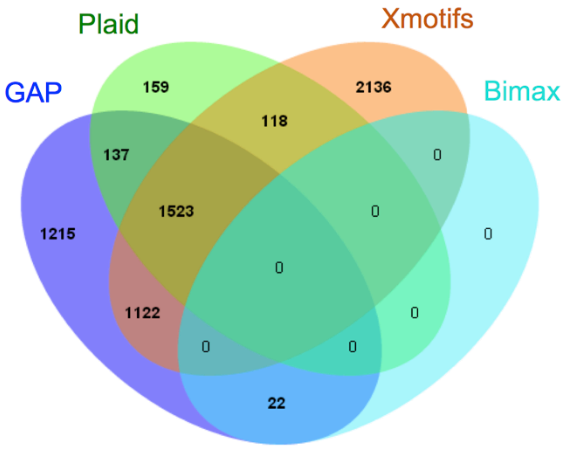

Figure S8. Coverage of species and domains from different bi-clustering methods. A) Statistics of bi-clustering results (number of clusters and coverage of species and protein domains) using different algorithms (GAP [1], Bimax [2], Plaid [3], and Xmotifs [4], the last three using the bi-clustering R toolbox of Kaiser and Leisch [5] with default settings). B) Venn diagram of the species clustered. C) Venn diagram of the protein domains clustered. Note that the large number (larger than that of GAP) of PDs that Xmotifs covered came from Bacteria, while many of the PDs from the other two superkingdoms covered by GAP were left out by Xmotifs.

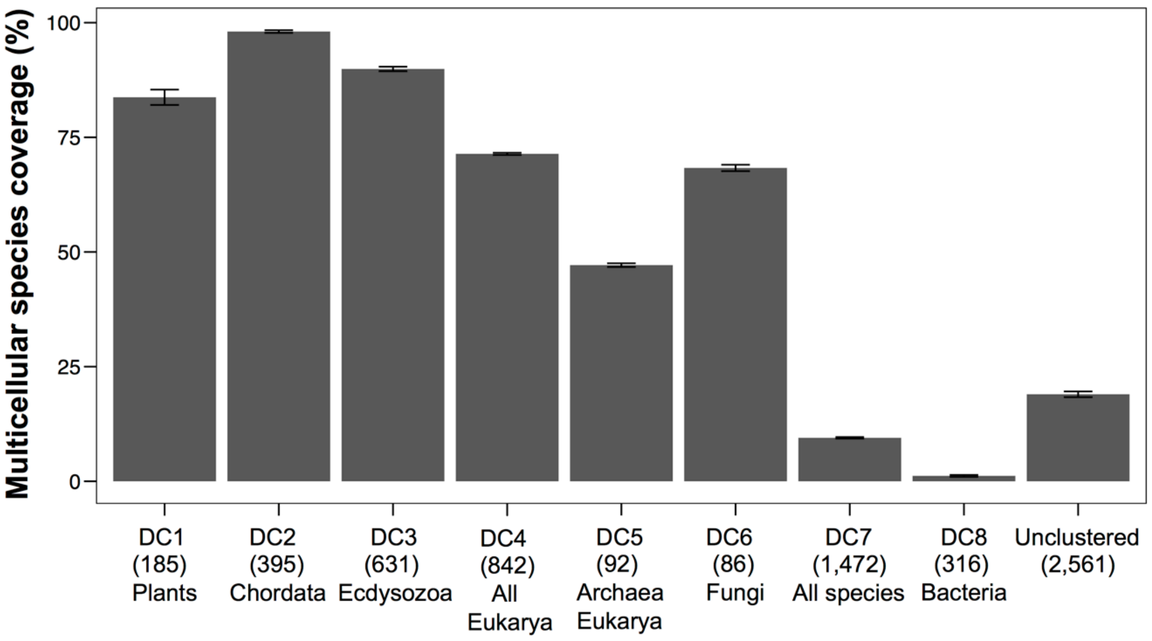

Figure S9. Multicellular species coverage of each of the eight PD clusters. A total of 133 Archaea species, 1,653 Bacteria species, and 348 Eukarya species were analyzed (see Methods). The Figure shows the averaged coverage of multicellular species in which a domain in a given PD cluster (or the collection of unclustered domains) was found to be present; the bar indicates the standard error.

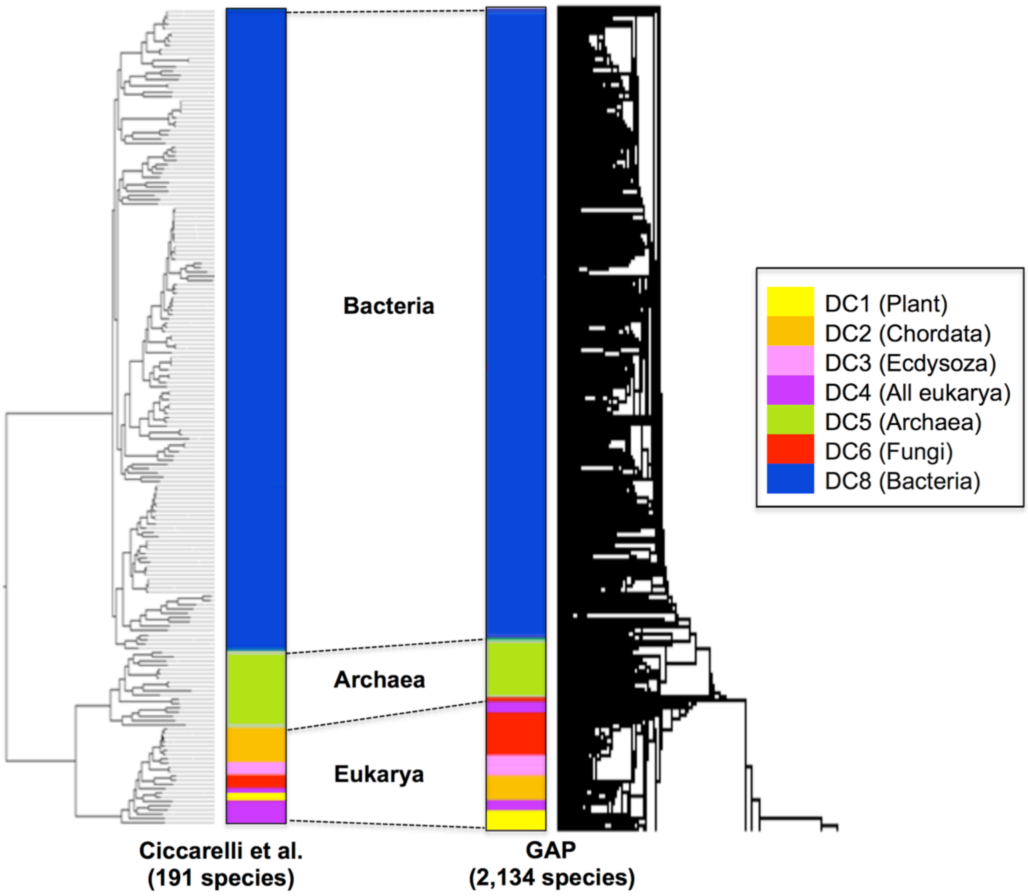

Figure S10. Comparison of a conventional phylogenetic tree with GAP clustering tree. The phylogenetic tree (left) is obtained from Ciccarelli, et al. [6], and the clustering tree (right) is generated by GAP. Generally speaking, the GAP clustering tree is mapped well to the phylogenetic tree. Therefore, our results might support the hypothesis proposed by Ciccarelli et al. that eukarya evolve from archaea. Note that the proportions of the species in the three superkingdoms are different between Ciccarelli et al. and our study, partly explaining the inclined dash lines that map the three superkingdoms of the two trees. The colors were coded according to that of the most dominant DC for a given species (see legend on the right).

References for Fig. S7, S8 and S10

[1]. Wu, H.M., Tien, Y. J. & Chen, C. H. GAP: A graphical environment for matrix visualization and cluster analysis. *Comput Stat Data An* **54**, 767-778 (2010).

[2]. Prelic, A., Bleuler, S., Zimmermann, P., et al. A systematic comparison and evaluation of biclustering methods for gene expression data. *Bioinformatics*, **22**(9), 1122–1129, (2006).

[3]. Lazzeroni, L. and Owen, A. Plaid models for gene expression data. *Statistica Sinica*, **12**, 61–86 (2002).

[4]. Murali, T. and Kasif, S. Extracting conserved gene expression motifs from gene expression. *Pacific Symposium on Biocomputing*, **8**, 77–88, (2003).

[5]. Kaiser, S. and Leisch, F. A toolbox for bicluster analysis in R. *Proceedings in Computational Statistics*, 201-208, Compstat 2008, Heidelberg, Germany, (2008).

[6]. Ciccarelli, F. D. *et al.* Toward automatic reconstruction of a highly resolved tree of life. *Science* **311**, 1283-1287 (2006).
